# Higher soil fauna abundance accelerates litter carbon release across an alpine forest-tundra ecotone

**DOI:** 10.1038/s41598-019-47072-0

**Published:** 2019-07-22

**Authors:** Yang Liu, Lifeng Wang, Runlian He, Yamei Chen, Zhenfeng Xu, Bo Tan, Li Zhang, Jiujin Xiao, Peng Zhu, Lianghua Chen, Li Guo, Jian Zhang

**Affiliations:** Long-term Research Station of Alpine Ecosystems, Key laboratory of Ecological Forestry Engineering of Sichuan Province, Institute of Ecology & Forests, Sichuan Agricultural University, Chengdu, 611130 China

**Keywords:** Forest ecology, Biodiversity

## Abstract

Upward shifts of alpine treelines and shrub expansion are occurring under climate change, and *Abies faxoniana* (AF) and *Rhododendron lapponicum* (RL) may become distributed at higher altitudes. How do abiotic factors and litter quality modulate the effects of soil fauna on carbon release in this context? A field decomposition experiment involving the foliar litter of AF and RL was conducted along an elevation gradient encompassing coniferous forest, alpine shrubland and alpine meadow by using litterbags with different mesh sizes (3 and 0.04 mm). The objective was to determine the influences of soil fauna, litter quality and abiotic factors on species-specific carbon release and their contributions during litter decomposition. Our findings demonstrated that higher soil fauna abundance and diversity facilitated litter carbon release. The contribution rates of soil fauna to carbon release (*C*_*fau*_) decreased with elevation increasing and decomposition time. *C*_*fau*_ are influenced by soil faunal diversity, dominant fauna groups (Collembola, Oribatida, Mesostigmata), and abiotic factors (temperature). Soil fauna significantly and directly regulated carbon release, abiotic factors indirectly regulated carbon release via altering soil fauna community composition and litter quality. This study improve our understanding in the mechanisms of decomposer contributions to carbon cycling in the context of global climate change.

## Introduction

Plant litter decomposition is a fundamental ecological process, linking nutrient cycling and energy flows in food webs and the structure and dynamics of ecosystems^1,2^. At the global scale, climate, litter quality and decomposers are considered the most important factors controlling litter decomposition^3^. Recent empirical studies have highlighted the need to explicitly acknowledge the role of decomposers, and it is believed that soil fauna enhance litter decomposition at both global and biome scales (average increment 27%)^3,4^. The soil fauna is an important component of ecosystems due to their functional roles in biogeochemical cycles in accelerating the rates of litter decomposition and nutrient transformation^5,6^.

Soil fauna play important roles in litter decomposition, mainly through the digestion of substrates, expansion of substrate surface area via fragmentation, and promotion of the rate of microbial inoculation into materials^1,7^. Direct increases in litter inputs to the soil or stimulation of microbial litter decomposition by soil fauna could have important implications for the formation of persistent soil organic matter and its C:N balance^8,9^. For example, the presence of isopods results in greater mobilization of various nutrients and litter-derived dissolved organic carbon, whereas the activity of Collembola has been found to increase the availability of litter-derived C to the soil microbial community^10^. Soil fauna generally exert a positive effect on the loss of litter mass^11,12^. However, accurate and quantitative predictions of the effects of soil fauna composition and diversity on C release remain lacking. In particular, the functional roles of soil fauna in alpine ecosystems are poorly understood.

Alpine ecosystems are considered thermally limited. However, due to human activities, average global temperature has increased, and treeline advance has occurred worldwide over the last century^13,14^. As part of global climate change, in alpine ecosystems, temperatures are increasing and precipitation regimes and patterns of snow cover are changing^15^. The plant community composition and diversity may shift accordingly, which is likely to influence litter mass loss and litter quality in the longer term^16^. Altered microclimates and associated changes in plant composition may decouple associations between plants and soil decomposer communities^17^. Therefore, the interactions between litter and decomposer communities in response to climate change appear to be uncertain. Although it is well understood how climate and substrate quality influence litter decomposition, the functional roles of decomposers in biogeochemical cycles remain unclear.

The alpine forest-tundra ecotone is the zone that extends from closed subalpine forest (timberline) to the upper boundary of the tree distribution. This ecotone typically appears as a patchy transition that consists of several intermediate vegetation zones and components, such as dark coniferous forest (CF), alpine shrubland (AS) and alpine meadow (AM)^18^. Litter decomposition in alpine forest-tundra ecotones is considered reduced relative to that at other lower altitudes due to thermal limitation. Previously, we studied the litter decomposition rates of representative plants (0.16–1.70) g/yr^19^ and the release of carbon and nutrients during litter decomposition^20,21^, mainly focusing on abiotic factors, such as freeze-thaw cycles, during the long snow-cover season. However, litter decomposition is also regulated by biotic factors^17^, and we previously found that soil fauna were more abundant in AS and CFs than in AMs across an alpine forest-tundra ecotone^22–24^. Additionally, we evaluated the contributions of soil fauna to lignin and cellulose degradation^25^. However, how soil fauna affect litter C release remains unclear.

There is a need to understand the roles of soil fauna in biogeochemical cycles in the context of global change. Thus, it is necessary to take soil fauna into account to adequately model the impacts of climate change on regional and global carbon dynamics^5^. Some research suggests that altitudinal gradients often produce climatic effects and influence the effects of soil fauna on decomposition^3,26^. Plant community composition, soil type and the micro-environment vary greatly across an alpine forest-tundra ecotone. We aimed to investigate how the contributions of soil fauna to decomposition are modulated by litter quality and abiotic factors. Treeline advance and shrub expansion into alpine ecosystems under climate change have been demonstrated^13,14^, which may led to upward shifts in the distributions of plant species, litter quality alterations, and variations in abiotic factors. Therefore, in this study, a field decomposition experiment involving foliar litter from *Abies faxoniana* and *Rhododendron lapponicum* was conducted along an elevational gradient encompassing CF, AS and AM using litterbags with different mesh sizes (3 and 0.04 mm). The objectives of our study were (1) to determine the abundance and species diversity of the soil fauna community and, more importantly, its contribution to C release during the decomposition of *A*. *faxoniana* and *R*. *lapponicum* foliar litter and (2) to explore how litter quality and abiotic factors modulate the effects of soil fauna on C release.

## Results

### Litter mass loss and carbon release

**Figure 1:**
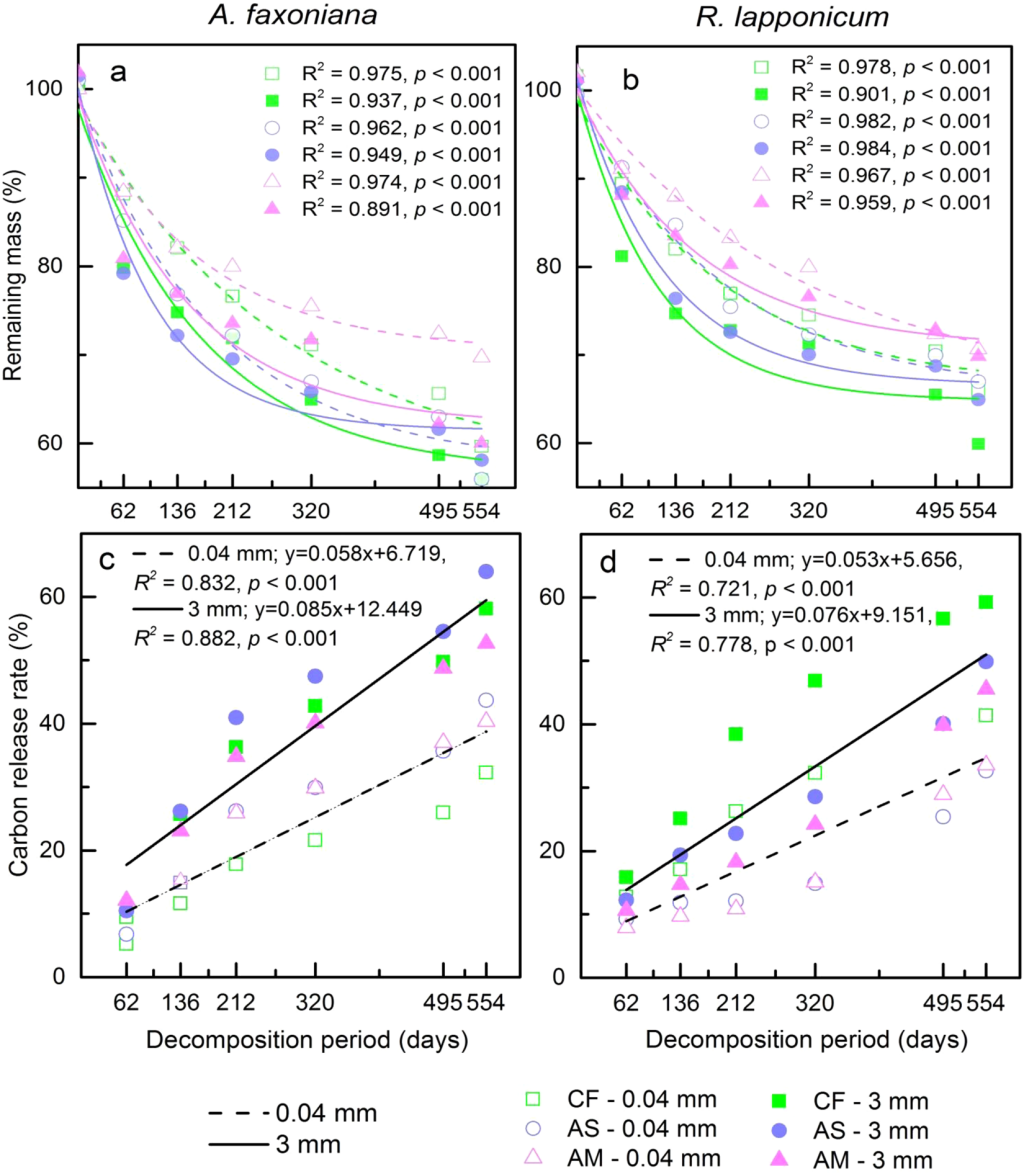
Regression plots of remaining mass and carbon release rate of *A*. *faxoniana* and *R*. *lapponicum* foliar litter with decomposition time across the forest-tundra ecotone. Different colours and different line styles represent curves fit within different litter decomposition treatments (n = 2). Unbroken and broken lines indicate litter with and without soil fauna, respectively, and the green, blue and pink symbols represent the remaining mass or carbon release rate in coniferous forest (CF), alpine shrubland (AS) and alpine meadow (AM), respectively.

The remaining litter mass exhibited a pattern of exponential decay over decomposition time. The mass loss from *A*. *faxoniana* foliar litter under the different treatments (3-mm and 0.04-mm meshes) was 44.46% and 40.44% in CF, 45.08% and 41.67% in AS, and 38.9% and 33.29% in AM (Fig. 1a), and the mass loss from *R*. *lapponicum* litter was 41.8% and 33.48% in CF, 36.22% and 35.63% in AS, and 31.51% and 30.43% in AM (Fig. 1b). The presence of soil fauna accelerated litter decomposition and carbon release in both types of litter (*P* < 0.05) (Table 1). The C release rates were significantly higher when soil fauna were involved in the decomposition of *A*. *faxoniana* (*R*^2^ = 0.882, *P < *0.001) and *R*. *lapponicum* (*R*^*2*^ = 0.778, *P < *0.001) litter than when soil fauna were excluded (Fig. 1c,d).

**Table 1:** Results of repeated-measures analysis of variance (ANOVA), including *F* values and significance for comparisons of remaining dry mass and carbon release rates at the three sites.

Site	Remaining mass rates				Carbon release rates			
	*df*	MS	*F*	*P*	*df*	MS	*F*	*P*
**Coniferous forest (CF)**								
Time	5	0.023	96.742	0.000	5	787.812	28.231	0.000
Treatment	1	0.015	50.612	0.000	1	1344.905	48.194	0.000
Time x treatment	5	0.000	1.362	0.086	5	45.224	3.621	0.022
**Alpine shrub (AS)**								
Time	5	0.031	18.278	0.000	5	757.258	10.009	0.001
Treatment	1	0.014	8.224	0.014	1	980.226	12.957	0.004
Time x treatment	5	0.000	1.104	0.099	5	32.119	1.425	0.082
**Alpine meadow (AM)**								
Time	5	0.023	10.153	0.001	5	657.818	11.918	0.000
Treatment	1	0.024	10.601	0.007	1	418.920	7.590	0.017
Time x treatment	5	0.000	1.179	0.097	5	12.700	1.230	0.094

### Soil fauna community composition and diversity

**Figure 2:**
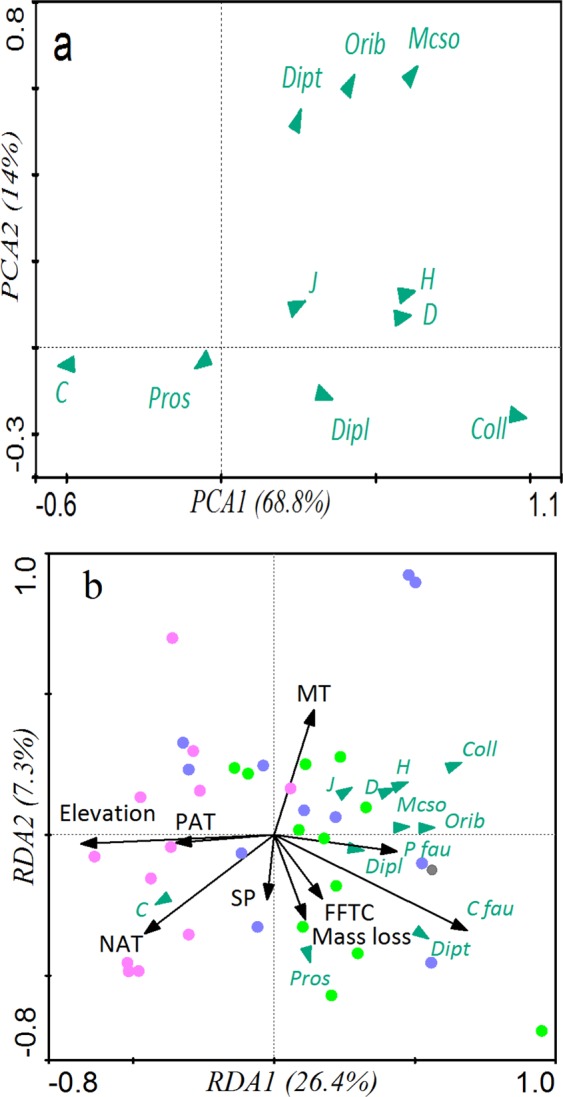
Principal component (**a**) and redundancy analysis (**b**) results of soil fauna diversity and abundance in association with selected physical and chemical properties during decomposition. Each point represents one sample of the soil fauna community from coniferous forest (green circles), alpine shrubland (blue circles), and alpine meadow (pink circles). Soil fauna community labels: Coll., Collembola; Ori., Oribatida; Meso., Mesostigmata; Pros., Prostigmata; Dipl., Diplura; Dipt., Diptera, *H*: Shannon-Wiener index, *J*: Pielou index, *C*: Simpson index, and *D*: Margalef index.

Across the entire ecotone, vegetation type and micro-environment significantly affected the composition of the soil fauna community (Fig. 2). *A*. *faxoniana* litter exhibited higher numbers of both soil fauna individuals and groups than did the *R*. *lapponicum* litter. A total of 1982 individuals representing 46 soil fauna groups were collected from the *A*. *faxoniana* litterbags, whereas the *R*. *lapponicum* litterbags contained 1272 individuals representing 36 groups. The highest number of soil fauna groups was found in CF, and the highest number of soil fauna individuals were observed in AS. The lowest numbers of both individuals and groups were found in AM. Collembola, Mesostigmata, Oribatid and Prostigmata were the predominant groups and accounted for 41.3%, 23.0%, 16.8% and 9.8%, respectively, of the total individuals in the *A*. *faxoniana* litter and 34.97%, 23.66%, 28.03% and 5.31%, respectively, of the total individuals in the *R*. *lapponicum* litter. Collembola was the most dominant component of the meso-fauna, and the number of individuals of this group decreased from CF to AM, whereas the numbers of Mesostigmata and Oribatid individuals increased from CF to AM. Isotomidae and Chironomidae were the only groups present in AS and CF (Tables S3, S4).

**Figure 3:**
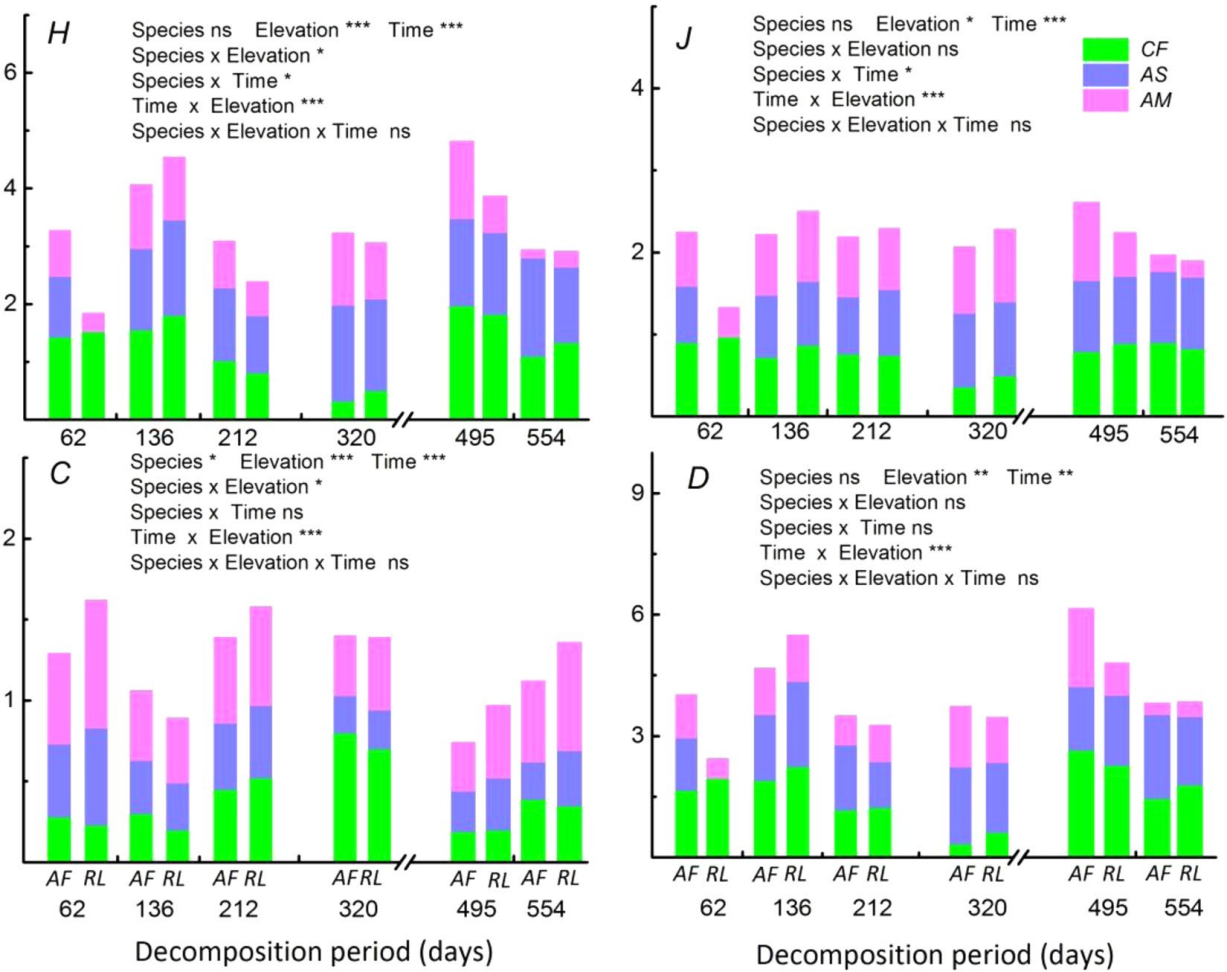
Diversity indices of soil fauna communities at three elevations in six key decomposition stages. *H*: Shannon-Wiener index, *J*: Pielou index, *C*: Simpson index, and *D*: Margalef index. Asterisks indicate statistical significance: *, **, and *** indicate significance at *P* < 0.05, *P* < 0.01, and *P* < 0.001, respectively; ns = not significant.

Vegetation type and decomposition time had significant effects on the diversity of the soil fauna communities (*H*, *J*, *C*, *D*) (Fig. 3). The value of the Shannon-Wiener, Pielou and Margalef indiceof the soil fauna were significantly higher in CF and AS than in AM, whereas the Simpson index values showed the opposite pattern. The PCA results revealed the relationship between soil fauna diversity and community composition during litter decomposition (Fig. 2a; Table 2). The cumulative contribution rate reached 82.8% (PC1: 68.8%, PC2: 14%), and the composition of the predominant group explained most of the variation in soil fauna diversity.

**Table 2:** Variance contributions of principal components analyses (PCA) and redundancy analyses (RDA).

	PCA			RDA	
	PRIN1	PRIN2		PRIN1	PRIN2
Collembola	0.987	−0.163	Elevation	−0.510	−0.201
Prostigmata	−0.085	−0.047	MT	0.106	0.298
Mesostigmata	0.636	0.648	FFTC	0.127	−0.152
Oribatida	0.431	0.636	SP	−0.183	−0.154
Diplura	0.360	−0.121	PAT	−0.260	−0.019
Diptera	0.259	0.549	NAT	−0.342	−0.236
Shannon-Wiener index (H)	0.627	0.128	Mass loss	0.083	−0.202
Pielou index (J)	0.271	0.105	*P*_*fau*_	0.325	−0.040
Simpson index (C)	−0.528	−0.040	*C*_*fau*_	0.510	−0.228
Margalef index (D)	0.614	0.072			
% of variance	0.688	0.140	% of variance	0.264	0.073
Cumulative (%)	68.8	82.8	Cumulative (%)	26.4	33.7

Note: Elevation: altitudinal gradient; MT: mean temperature; FFTC: frequency of freeze-thawing cycles; SP: depth of snow cover; PAT: positive accumulated temperature; NAT: negative accumulated temperature

### Contribution of soil fauna to C release

**Figure 4:**
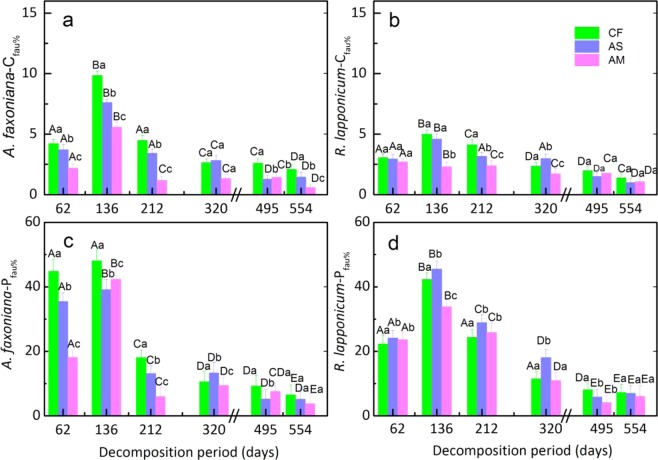
The contribution percentage (%) of soil fauna to carbon release in different decomposition stages (mean ± SE, n = 5). (**a**) *A*. *faxoniana* − *C*_*fau*_; (**b**) *R*. *lapponicum* − *C*_*fau*_; (**c**) *A*. *faxoniana* − *P*_*fau*_; (**d**) *R*. *lapponicum* - *P*_*fau*_. Different uppercase letters indicate significant differences (*P* < 0.05) among decomposition stages at the same elevation within a species. Different lowercase letters indicate significant differences (*P < *0.05) among elevations within the same decomposition stage and within a species.

**Figure 5:**
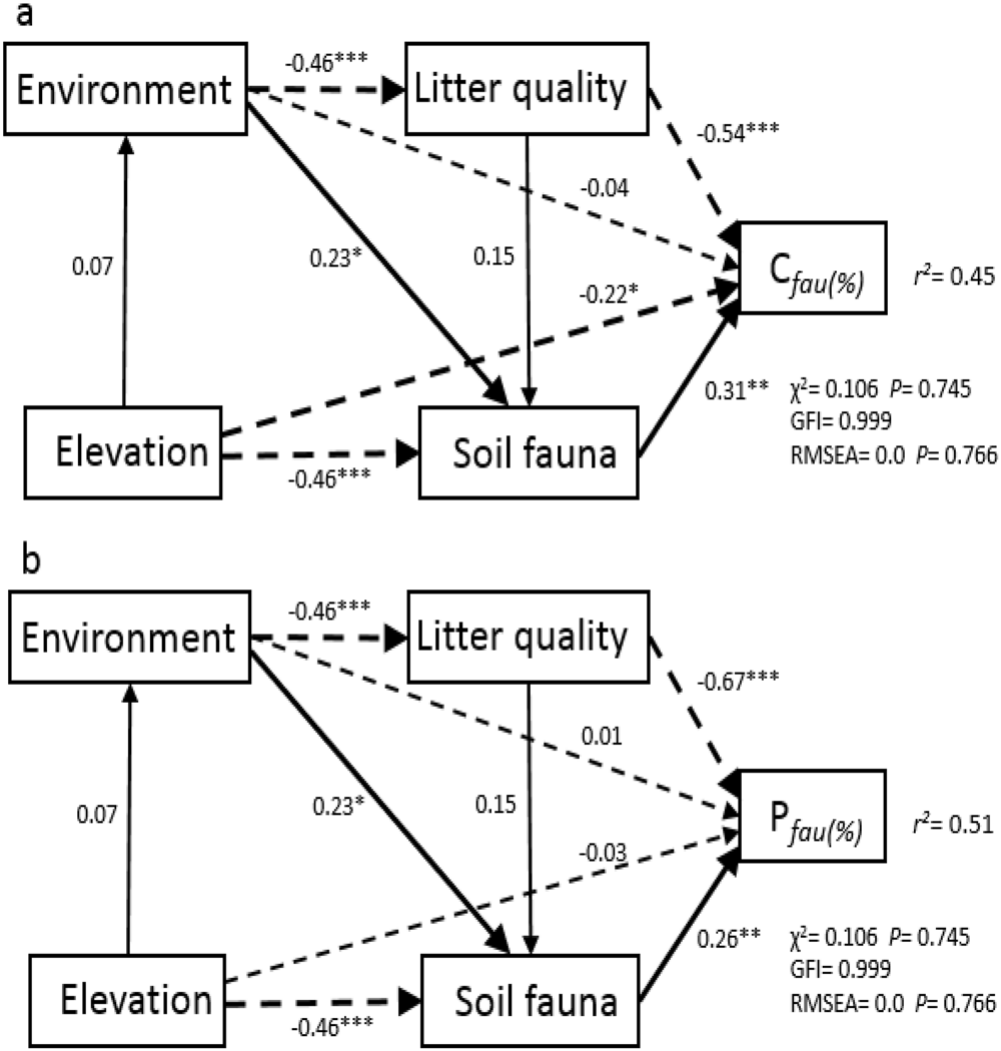
Structural equation models describing the influences of environmental conditions, soil fauna community composition and litter quality on carbon loss (**a**) and the contribution of soil fauna to carbon loss (**b**). Note that two environmental conditions and litter quality components of the PCA were included in the SEM because both had eigenvalues > 1. Pearson correlations among soil fauna variables, environmental conditions, and litter quality variables and their corresponding PC axes. Soil fauna variables, PC1: Eigenvalues1 = 2.1; Coll = 0.69**; Pros = −0.01; Mcso = 0.63**; Orib = 0.48**; Dipl = 0.17; Dipt = 0.28; *H* = 0.97**; *J* = 0.77**; *C* = −0.89**; *D* = 0.95**. Environmental factors, PC1: Eigenvalues1 = 1.58; MT = 0.84**, FFTC = −0.54**, PAT = 0.56**, NAT = 0.71**, SP = −0.82**; PC2: Eigenvalues2 = 1.04; MT = 0.37*, FFTC = −0.72**, PAT = −0.45**, NAT = −0.44**, SP = 0.16; Litter quality model, PC1: Eigenvalues1 = 1.64; C = 0.58, N = −0.88*, P = −0.97**, Lignin = 0.45, Cellulose = −0.49; PC2: Eigenvalues2 = 1.19; C = −0.54, N = −0.41, P = −0.17, Lignin = −0.88*,Cellulose = 0.61. Solid and dotted lines denote positive and negative effects, respectively, and a bold line denotes a significantly relationship. Asterisks indicate statistical significance: *, **, and *** indicate significance at *P* < 0.05, *P* < 0.01, and *P* < 0.001, respectively.

Over the entire field incubation period, the C release rates from *A*. *faxoniana* litter decomposition driven by soil fauna (*C*_*fau*_) in CF, AS and AM were 25.86%, 20.23% and 12.3% (Fig. 4a), respectively, whereas those for *R*. *lapponicum* litter were 17.84%, 16.22% and 11.96%, respectively (Fig. 4b). The daily average C release (*P*_*fau*_) contributions of the soil fauna during *A*. *faxoniana* litter decomposition in CF, AS and AM were 0.25%, 0.2% and 0.16% respectively (Fig. 4c), and those during *R*. *lapponicum* litter decomposition were 0.21%, 0.23% and 0.19%, respectively (Fig. 4d). Soil fauna (0.31, *P* < 0.01) and litter quality (−0.54, *P* < 0.001) significantly and directly affected C release during decomposition (Fig. 5a) and the C contribution of soil fauna (Fig. 5b). C release was significantly higher in *A*. *faxoniana* litter than in *R*. *lapponicum* litter, which was mainly due to differences between the two types of litter in N, P concentration. Abiotic factors indirectly regulated C release via altering the temporal dynamics of the soil fauna community and litter quality (Fig. 5a,b), which decreased from CF to AM (*P < *0.05) for both species. *C*_*fau*_ and *P*_*fau*_ were highest in the early decomposition stages and decreased with increasing decomposition time. However, *C*_*fau*_ and *P*_*fau*_ showed different patterns over the course of decomposition between the two litter species, with the *C*_*fau*_ and *P*_*fau*_ of *A*. *faxoniana* litter decreasing from 136 days onward and those of *R*. *lapponicum* litter decreasing from 212 days onward.

Redundancy analysis (RDA) revealed the mass loss and C release rates driven by soil fauna in association with soil faunal diversity and abiotic factors during decomposition. These factors explained 33.7% of the total variance in the composition of soil fauna community (Fig. 2b; Table 2). The first canonical axis was mainly determined by *C*_*fau*_, elevation and NAT, which together explained 26.4% of the total variation, whereas the second canonical axis explained 7.3% of the total variation and included mean temperature (MT: eigenvector of 0.3). Similarly, in the SEM analysis (Fig. 5), the presence of soil fauna had a significant effect on C release, and *C*_*fau*_ and *P*_*fau*_ displayed positive relationships with each of the soil fauna diversity indices (Fig. 5; *H* = 0.97, *J* = 0.77, *C* = *−*0.89, *D* = 0.95; all *P* < 0.01) and dominant groups (Collembola: 0.69 = ;, Oribatida: 0.48, Mesostigmata: 0.63; all *P* < 0.01).

## Discussion

### Soil fauna accelerate litter carbon release

Plant litter carbon release is an important biogeochemical process in terrestrial ecosystems^27^. The extensive manipulation experiment by Wall *et al*.^5^ indicated that soil fauna significantly increases litter mass loss in most biomes. Soil fauna have been found to enhance litter decomposition (average increment ~27%) at both global and biome scales^3^, but little is known about their impact on carbon release during litter decomposition and their influence on ecosystem carbon cycling. Our findings indicated that litter mass loss and C release rates were significantly accelerated when soil fauna were allowed access to the litter. We integrated the contribution of soil fauna into models of carbon release (Fig. 5), and the structural equation models indicated that soil fauna had direct significant positive effects on *C*_*fau*_ and *P*_*fau*_ (0.31 and 0.26, all *P* < 0.01). Inclusion of biome-specific soil fauna effects on litter decomposition is advocated as a means to reduce the unexplained variation in large-scale decomposition models^3^. According to the meta-analysis of Frouz *et al*.^28^, only part of the litter consumed by soil fauna is assimilated; the rest is returned to the soil as faeces. The percentage of food that is assimilated varies substantially among various groups of soil macrofauna.

Faunal activity accelerated the removal of litter from the litter layer but had no effect on overall mineralization because most of the removed material was stored in the mineral layer. Soil fauna can cause physical fragmentation, stimulation of microbial activity and transportation of fungal and bacterial propagules, thereby promoting C release due to litter decay^29^. By feeding on litter, soil fauna typically redistribute organic matter in the soil profile by modifying the leaching or movement of particulate organic matter. Fauna may affect the amount and quality of dissolved organic matter entering the soil from decomposing litter and modify the priming effect. In addition, soil fauna can increase the release of DOC by accelerating the turnover of microbial biomass^30^. DOC can be rapidly leached from litter bags and then used by soil microorganisms outside the bags, and a portion of the C that disappears from litter bags is released into the atmosphere through the respiration of soil fauna and microorganisms. Our study revealed that soil fauna can promote carbon release in the preliminary stage of decomposition, especially within the first 212 decay days. A meta-analysis revealed that microarthropods generally exert a positive effect on litter mass loss^12^. Many other studies have similarly indicated that soil fauna consume and break up litter in the early decomposition stages. For example, Collembola directly contributes to C transport in the litter-soil environment^8^, and Prostigmata is an initial coloniser during litter decomposition and is usually present in the early stages of decomposition^31^. In contrast, Oribatida shows a preference for later stages of decomposition. However, Collembola significantly increases the mineralization of SOC at the late stage of incubation, when litter labile C is depleted, although there is no evidence that Collembola affects the litter decomposition rate or microbial biomass^32^. In contrast, soil fauna were shown to support ecosystem productivity and soil carbon accrual in tallgrass prairie, where microarthropod suppression slowed litter mass loss and decreased the litter carbon input into the soil during the first 18 months of decomposition^4^. Therefore, soil fauna simultaneously promote not only the removal of litter from soil surface but also the accumulation of their faeces and fragmented litter in soil^30^.

In this study, soil fauna community abundance and diversity were the primary factors affecting carbon release. The PCA and RDA analyses indicated that *C*_*fau*_ and *P*_*fau*_ displayed positive relationships with the soil fauna diversity indices (Fig. 5; *H*, *J*, *C*, *D*) and dominant groups (*P* < 0.01). Similarly, mass loss was significantly correlated with the abundance of groups such as total Acari, Collembolans, and Mesostigmata mites. The faunal contribution to decomposition varies markedly among environmental gradients that differ in litter faunal diversity^26^. Correlational research has revealed strong impacts of soil fauna on carbon turnover^27^; these fauna include not only earthworms and other macrofauna but also microfauna such as Collembola. For example, introduced earthworms can double microaggregate formation and substantially stabilise new C in topsoil^33,34^. The presence of a single Collembola species may enhance microbial biomass by 56%^35^, and litter decay rates can be increased by up to 30% due to Collembola grazing^36^. In this study, we analyzed only microarthropod community taxa. Other soil fauna groups, such as Anelida, Enchytraeidae and earthworms, may play important roles in decomposition even if they (particularly Enchytraeidae) are not detected.

### Litter quality control carbon release

Litter quality is a primary determinant of the resistance of litter to decomposition^1^. The initial chemical composition of litter and the chemical changes that occur during decomposition directly affect carbon release rates at large scales. Furthermore, litter quantity and quality affect the structure and functions of soil communities^37^, which indirectly affect carbon release. Whether litter quality is a significant determinant of litter decomposition depends on both the soil community composition and the length of field exposure because decomposition rate is dependent on mobility. Previous research found that soil organisms of all size classes responded positively to increased litter quality^38^. In the present study, the numbers of individuals and groups of soil fauna in *A*. *faxoniana* litter were higher than those in *R*. *lapponicum* litter. This difference mainly occurred because soil fauna favour litter with low C/N and lignin/N ratios (Hättenschwiler *et al*.^2^; Bradford *et al*.^17^; Berg *et al*., 1993). Because the initial chemical composition of *R*. *lapponicum* was more resistant to the decomposition process than was that of *A*. *faxoniana* litter (Table S2), the impact of soil fauna on C release was greater for *A*. *faxoniana* litter than for *R*. *lapponicum* litter. Litter species identity is an important determinant of the abundance and diversity of soil fauna^2,39^. We found that litter quality had significant negative effects on *C*_*fau*_ and *P*_*fau*_ (−0.54 and −0.67, *P* < 0.001) in the structural equation models (Fig. 5), which highlights the importance of the relationship between litter quality and decomposition. At the regional scale, litter chemistry becomes the most important determinant of litter decomposition rate. In the context of treeline advance or tundra expansion, plant species shifts along elevation lead to variations in plant litter composition that affect the interaction of litter species-specific decomposition and decomposer diversity. Therefore, the mechanisms of litter decomposition and C transformation into soil in alpine ecosystems under climate change are complicated.

### The effects of abiotic factors on carbon release

We found that the decomposition rate (*k*) of *A*. *faxoniana* and *R*. *lapponicum* was 0.209–0.243 yr^−1^ and 0.173–0.189 yr^−1^, respectively; representing on average 43% and 35% of the total decomposition within 554 days. These results indicate that a long time is required for these litters to decay completely in alpine areas subjected to to thermal limitation. RDA and SEM analyses indicated that elevation, NAT and MT were the main abiotic factors affecting *C*_*fau*_ *and P*_*fau*_ (Fig. 2). In addition, SEM indicated that elevation had direct significant effects on the soil fauna communities (−0.46, all *P* < 0.001) and that abiotic factors had direct significant effects on both litter quality and soil fauna community (−0.46 and 0.23, all *P* < 0.01). All of the soil fauna diversity indices and dominant groups positively related to mean temperature and negatively relate to elevation.

We found that the soil macro-faunal communities of alpine shrubland and coniferous forest were more similar to each other than to the macro-faunal communities of alpine meadow. The dominant macro-faunal groups of alpine meadow (Lumbricida, Formicidae, Scarabaeidae, Bibionidae) differed from those of forest and shrubland (Staphylinidae, Lithobiida, Geophilomorph). However, meso-faunal groups (Nematoda, Enchytraeidae, Eutardigrada, Isotomidae, Harpacticoida Oribatuloidae) have been shown to be similar among these three vegetation types^23^. This is not only because the vegetation communities and soil type are more similar between alpine shrubland and coniferous forest than between alpine meadow but also because abiotic factors, such as MT and NAT, are more similar between alpine shrubland and coniferous forest.

Home-field advantage (HFA) is the notion that plant litter decomposes more rapidly underneath plants from its home environment than underneath plants from other areas. Litter mass loss has been shown to be, on average, 8% more rapid at home than away^40–42^. We speculate that HFA exists in the studied alpine forest-tundra ecotone, as *A*. *faxoniana* and *R*. *lapponicum* were the predominant species in CF and AS, respectively. Decomposer communities are specialized to break down litter from the plants they associate with^43^; this specialization may be related to differences in the abundance of some faunal functional groups or to the species-specific trophic behaviour of soil fauna^41^. We suggest that the magnitude of HFA effect on litter mass loss may be underestimated, since litterbags restrict the removal of litter from litterbags by soil fauna, and that HFA may increase with greater differences in litter quality between two litter types. Moreover, HFA could potentially be disrupted by timberline advance or tundra expansion^42^. According to the present results, if the distributions of *A*. *faxoniana* and *R*. *lapponicum* shift with elevation, soil faunas will slow down the litter C release at a higher altitudes. Hence, an altered climate and associated changes in vegetation composition may decouple the associations between plants and soil decomposer communities^1,44^, with consequences for ecosystem processes such as decomposition and carbon cycling. Furthermore, the impacts of climate change on decomposition are likely to depend on the local compositions of the fungal and invertebrate decomposer communities^36^.

## Conclusion

Litter decomposition is known to generally be controlled by climate, litter quality and decomposer characteristics^5,15^, but the relative importance of these drivers can differ among scales and ecosystems. This study highlighted the important roles of soil fauna in litter decomposition, and the findings support the hypothesis that higher soil fauna abundance and diversity facilitate litter carbon release across an alpine forest-tundra ecotone. Furthermore, the findings provide insight into the relative importance of abiotic factors, litter quality and soil fauna in influencing litter carbon release rates and on how abiotic factors and litter quality modulate the effects of soil fauna on such rates. The evaluation of biotic factors that control litter decomposition has important implications for the long-term functioning of carbon sequestration. The relationship between decomposition and soil fauna with timberline advance or tundra expansion under climate change deserves further attention. More long-term studies on soil faunal control of carbon release during decomposition in alpine ecosystems are needed.

## Materials and Methods

### Site description

The study was conducted at the Long-term Research Station of Alpine Forest Ecosystems (longitude, 102°41′32″ to 102°41′54″E; latitude, 31°51′48″ to 31°51′53″N; approximately 3900–4200 m above sea level (a.s.l.), with pronounced vertical zonality), which is located in the Miyaluo Nature Reserve, Sichuan Province, southwest China. The study area is a transitional zone situated between the Tibetan Plateau and the Sichuan Basin, and it is covered by mixed coniferous broadleaved forests, dark coniferous forest, alpine shrubland and alpine meadow from the valley to the ridge, with a snow belt above 4500 m a.s.l. The weather is cool in summer and cold in winter. The annual mean air temperature ranges from approximately 6–12 °C, and the absolute maximum and minimum air temperatures are 12.6 °C in July and −8 °C in January, respectively. In the alpine zone, snow-cover season begins in November and lasts until the end of April (approximately 6 or 7 months).

Three 50-m-wide transects, which were separated by more than 1 km, were established perpendicular to the contour line in the forest-tundra ecotone. The vegetation types in these three transects, along which permanent sample plots were established in 2008, vary from CF (3900–4000 m a.s.l.) to AS (4000–4200 m a.s.l.) to AM (>4200 m a.s.l.) (Fig. S1). The dominant tree species in the CF and AS are *A*. *faxoniana* and *R*. *lapponicum*, respectively. The upward shifts of the alpine treeline and shrub expansion may be leading to shifts in *A*. *faxoniana* and *R*. *lapponicum* distributions toward higher altitude. The forest understory is cold and wet, with thick moss and humus layers, and the coverage and height of the woody shrubs decrease from the shrubland to the meadow. The shrubland is primarily characterized by vegetation consisting of woody shrub species and long-lived perennial herbaceous plants, and the alpine treeline, which is located near the upper elevational boundary of CF, is at approximately 4000 m a.s.l.^20^. The soils are Cryumbreps and Histosols (United States Department of Agriculture Soil Taxonomy), and soil subsamples were sent to laboratory for standard soil physicochemical analyses (Table S1).

### Experimental design and sampling

Freshly fallen leaf litter from two woody species (*A*. *faxoniana* and *R*. *lapponicum*) was collected in October 2012 across the alpine forest-tundra ecotone. These two plant types represent the predominant tree and shrub species in the area. To avoid structural damage to the litter during oven drying, the freshly fallen litter was first air dried for more than two weeks at room temperature. The contribution of the soil fauna to C release via litter decomposition was determined using litterbags of two different mesh sizes^45^. Samples of 10 g each were placed in 20 × 20 cm nylon litterbags with a mesh size of 0.04 mm on the bottom and a mesh size of either 0.04 mm (to exclude soil fauna) or 3.00 mm (to allow soil fauna to enter) on the top. A total of 370 litterbags (two species × two mesh types × three elevations × five replicates × six sampling dates + 10 samples of variable initial quality) were placed throughout the forest-tundra ecotone. Subsamples of the foliar litter from each species were oven dried at 65 °C for 72 h to determine their initial quality (Table S2).

All litter in each litterbag was spread as flat as possible to maintain contact with the soil, and each litterbag was labelled with an aluminium tag to identify the corresponding treatment. Button temperature dataloggers (iButton DS1921, Maxim/Dallas Semiconductor, Sunnyvale, CA, USA) were wrapped in plastic to protect them from moisture and then placed inside the litterbags to automatically monitor the temperature every 3 h. The processes of freezing and thawing can be viewed as the accumulation and release of thermal energy^46^; therefore, the sampling dates were selected based on changes in the freezing and thawing dynamics determined from previous field observations^20^. Litterbags were randomly sampled from each sample plot on 4 July 2013, 7 September 2013, 23 November 2013, 12 March 2014, 4 September 2014, and 4 November 2014.

A freeze-thaw cycle was defined as a decrease in temperature to below 0 °C and maintenance of sub-zero temperatures for at least 3 h followed by an increase to above 0 °C and maintenance of above-zero temperatures for at least 3 h, and vice versa^47^. The daily mean temperatures (MTs) for each stage were calculated separately (Fig. S2). To better characterize the temperature changes, the positive accumulated temperature (PAT) and negative accumulated temperature (NAT) were calculated. In addition, the depth of the snow cover at each elevation was measured.

### Fauna inventory

Data were analysed at the Laboratory of Ecological Forestry Engineering of Sichuan Province of the Institute of Ecology and Forests, Sichuan Agricultural University, Chengdu, China. The collected litterbags were immediately placed in modified Tullgren extractors to remove litter invertebrates^48^; this collection strategy requires convenient access to the sites and efficient extraction techniques. All extracted faunal samples were preserved in 75% ethanol and then sorted under a dissecting microscope into broad taxonomic groups; the Pic torial Keys for the Soil Animals of China were used to identify each sample to the family level^49,50^, and the taxonomic identification was wide applied in many studies^26,39^.

### Litter chemistry analyses

After separation, the surface debris was removed from the collected litterbags, and the litter inside was oven dried at 65 °C to a constant weight. After weighing, all the samples were ground and milled for chemistry analyses. Carbon content was determined using the dichromate oxidation ferrous sulfate titration method. Nitrogen and phosphorus contents were determined using the Kjeldahl method and molybdenum-blue colorimetric method, respectively. Total phenolic content was measured using the Folin-Ciocalteu method. Cellulose and lignin concentrations were measured using the Van Soest method^25^.

### Calculations and statistical analyses

For the foliar litter in each type of litterbag, C release rates (*L*_*C*_) throughout the one and a half years of the litter decomposition experiment were calculated, and the release rates due to soil fauna in each period (*C*_*fau*_) were determined. The contribution of the soil fauna to C release associated with litter decomposition for each period (*P*_*fau*_) was calculated as follows:

$${{L}}_{{C}}({ \% })={(}{{M}}_{{0}}{{C}}_{{0}}{-}{{M}}_{{i}}{{C}}_{{i}}{)}/{{M}}_{{0}}{{C}}_{{0}}\times {100}$$

$${C}_{fau}( \% )=({L}_{lt}-{L}_{st})-({L}_{l(t-1)}-{L}_{s(t-1)})$$

$${P}_{fau}( \% )={C}_{fau}/({L}_{lt}-{L}_{s(t-1)})\times 100$$

where M_0_ is the initial oven-dried litter mass (g); M_i_ is the dry mass of the litter remaining in the bag in each sampling period (g); C_0_ is the initial concentration (*g·kg*^−1^); C_*i*_ is the concentration of C in the remaining litter in each sampling period; (L_lt_ − L_st_) is the difference in the C release rate between the two litterbag mesh sizes for sampling date “t”; (L_*l*(t-1)_ − L_s(t−1)_) is the difference in the mass loss rate between the two litterbag mesh sizes for sampling date “(t - 1)” (t = 1, 2, 3, 4, 5, 6); L_lt_ and L_s(t-1)_ are the C release rates for the large-mesh-size litterbags on sampling dates “t” and “(t-1)” (t = 1, 2, 3, 4, 5, 6), respectively; and L_l_ and L_s_ are the C release loss rates for the large- and small-mesh-size litterbags, respectively, on the last sampling date.

The Shannon-Wiener diversity index (H), Pielou evenness index (J), Simpson diversity index (C) and Margalef richness index (D) were calculated for the soil faunal community as follows:

$$H=-\sum _{i=1}^{S}P{\rm{i}}\,\mathrm{ln}\,Pi$$

$$C=\sum {(Pi)}^{2}$$

$$J=H/l{n}_{s}$$

$$D=(S-1)/ln\,N$$

where *P*_*i*_ is the relative percentage of the soil fauna of type “i” in each sampling period; *S* is the number of groups; and *N* is the total number of individuals in the soil fauna.

Independent samples t-tests were used to evaluate differences in the initial substrates between the two plant species, with an alpha level of 0.05. Repeated measures analysis of variance was performed to test for variations in mass loss and C release rate with treatment and elevational gradient over the course of litter decay, which were evaluated via exponential and linear regression during different key periods. In addition, repeated measures analysis of variance was used to evaluate carbon release rate (*C*_*fau*_) and the contribution ratio of carbon release (*P*_*fau*_) due to soil fauna during litter decomposition. Three-way nested analysis of variance was used to evaluate the effects of litter species, elevation and time on the diversity indices of soil fauna communities.

Principal component analysis (PCA) was employed to evaluate the effects of litter quality on the composition of the soil fauna community, and11 factors were considered. These factors included the Shannon-Wiener index (H), Pielou index (J), Simpson index (C), Margalef index (D), and the dominant soil fauna groups: Collembola, Oribatida, Mesostigmata, Prostigmata, Diplura, Diptera. and Psocoptera (which together accounted for more than 95% of the total abundance). Redundancy analysis (RDA) was used to analyze the relationships between soil fauna communities and litter decomposition. The analysis included two sets of variables: abiotic factors related to local site conditions (Elevation, MT, FFTC, PAT, NAT, SP) and litter decomposition variables (mass loss, *C*_*fau*_, *P*_*fau*_)^31^. We explored the influences of local abiotic factors, soil fauna community composition and litter quality traits on the contribution of soil fauna to C release using structural equation modelling^3,7^. We selected C, N, P, lignin, cellulose and phenol as indicators of litter quality^20,25^. Before building the model, we reduced the dimensionality of the environmental variables and litter quality data set by generating multivariate axes of variability suitable for use in SEM. PCA was also used to capture most of the variance due to environmental factors and litter quality, and only PCA axes with eigenvalues >1 were retained7. It was necessary to transform the measured data using log (x + 1). The overall goodness-of-fit of each model was evaluated by the χ^2^ goodness-of-fit test, the root mean square error of approximation index (RMSEA) and the goodness-of-fit index (GFI). Statistical analyses were performed with SPSS version 23.0 and AMOS version 23.0 (SPSS Inc., Chicago, IL, USA).

## Supplementary information

Higher soil fauna abundance accelerates litter carbon release across an alpine forest-tundra ecotone
