# Supplementary material for: Higher soil fauna abundance accelerates litter carbon release across an alpine forest-tundra ecotone
Source: Sci Rep. 2019 Jul 22;9:10561. doi: 10.1038/s41598-019-47072-0 (PMC6646657; doi:10.1038/s41598-019-47072-0)
Supplement: Supplementary file 1 — Higher soil fauna abundance accelerates litter carbon release across an alpine forest-tundra ecotone [file 41598_2019_47072_MOESM1_ESM.docx]

**Title:** **Higher soil fauna abundance accelerates litter carbon release** **across an alpine forest-tundra ecotone**

Authors: Yang Liu^&^, Lifeng Wang^&^, Runlian He^&^, Yamei Chen, Zhenfeng Xu, Bo Tan, Li Zhang, Jiujin Xiao, Peng Zhu, Lianghua Chen, Li Guo, Jian Zhang*

**Addresses:** Long-term Research Station of Alpine Ecosystems, Key laboratory of Ecological Forestry Engineering of Sichuan Province, Institute of Ecology & Forests, Sichuan Agricultural University, Chengdu 611130, China

* **Corresponding author;**

E-mail address: sicauzhangjian@163.com

Telephone: +8619940876810

^&^ These authors contributed equally to the paper.


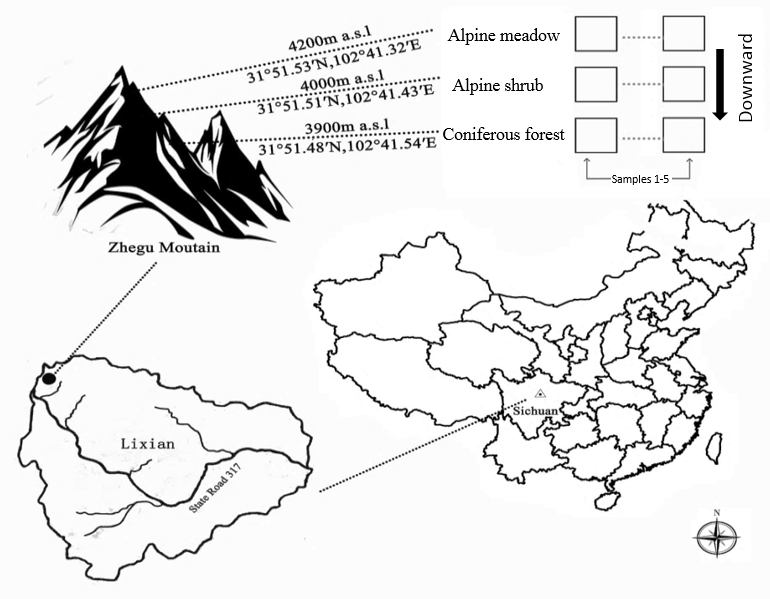


Fig. S1 Location of sampling sites on Zhegu Mountain, western Sichuan Province, China.

The three sampling sites were located along an elevation gradient from 3900 to 4200 m with similar slope and directions. CF: coniferous forest; AS: alpine shrub; AM: alpine meadow.


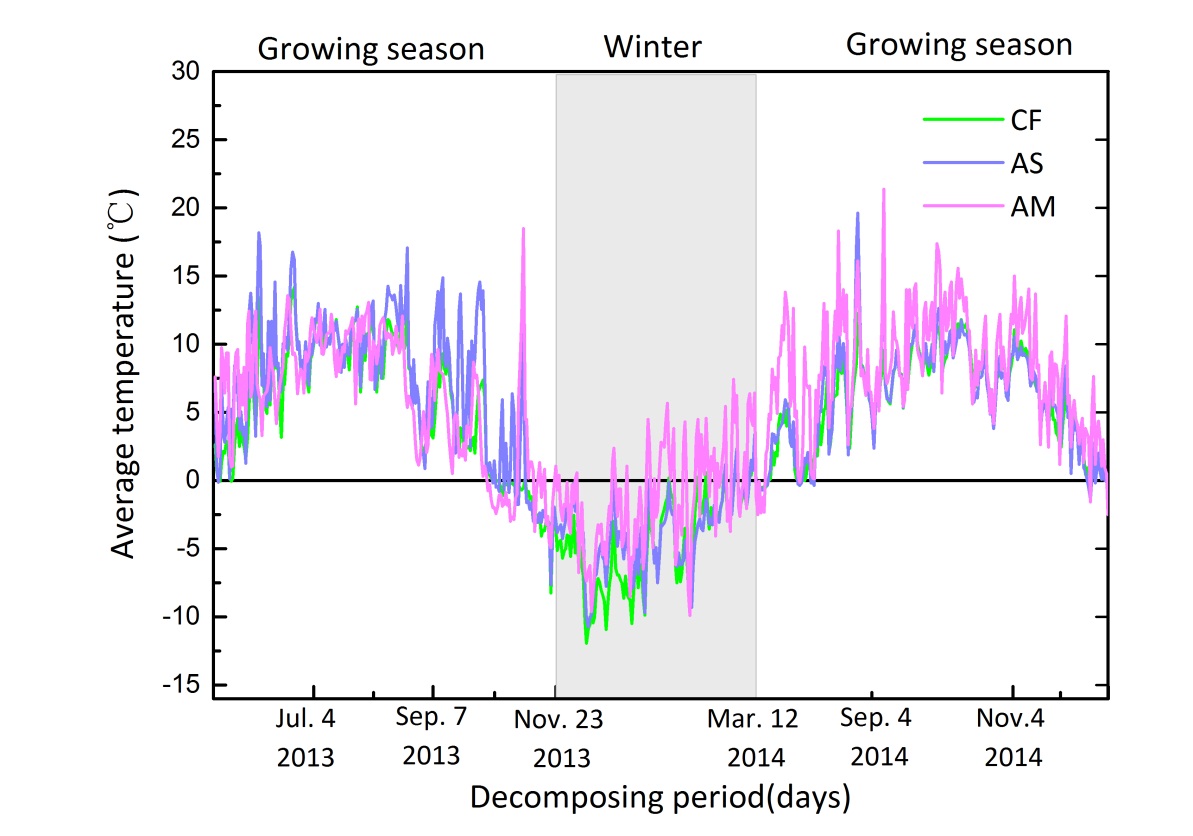


Fig. S2 Daily mean temperature in the litterbags at different elevations (a total of 554 days of exposure in the field). Sampling stages were partitioned based on differences in freezing and thawing as the temperature changed. CF: coniferous forest; AS: alpine shrub; AM: alpine meadow.

Table S1 [Physical and chemical properties](javascript:;) of the soil (0-20 cm) in an alpine forest-tundra ecotone.

| Soil Property | Coniferous forest  (CF) | Alpine shrub  (AS) | Alpine meadow  (AM) |
| --- | --- | --- | --- |
| Soil moisture (%) | 34.3 | 35.08 | 31.46 |
| Soil bulk density (g cm^-3^) | 0.9 | 0.83 | 0.88 |
| Soil total porosity (%) | 64.5 | 67.56 | 61.5 |
| Soil capillary pores (%) | 52.21 | 50.44 | 48.14 |
| Soil non-capillary pores (%) | 12.29 | 17.12 | 13.36 |
| Soil pH | 5.04 ± 0.06 | 4.97 ± 0.03 | 4.88 ± 0.03 |
| Soil SOC (g kg^-1^) | 84.13 ± 4.91 | 78.42 ± 0.09 | 57.48 ± 0.01 |
| Soil TN (g kg^-1^) | 4. 4 ± 0.03 | 3.34 ± 0.11 | 2.33 ± 0.07 |
| Soil TP (g kg^-1^) | 0.98 ± 0.01 | 0.79 ± 0.01 | 0.73 ± 0.02 |
| Soil TK (g kg^-1^) | 17.87 ± 0.32 | 21.17 ± 0.23 | 19.68 ± 0.77 |
| Soil available N (mg kg^-1^) | 551.46 ± 17.17 | 450.74 ± 3.94 | 229.87 ± 1.29 |
| Soil available P (mg kg^-1^) | 2.25 ± 0.05 | 4.53 ± 0.03 | 16.19 ± 0.45 |
| Soil available K (mg kg^-1^) | 449.77 ± 0.65 | 228.51 ± 5.42 | 147.33 ± 4.37 |

Table S2 Litter chemistry of *Abies faxoniana* and *Rhododendron lapponicum* foliar litter expressed as potential litter quality variables (mean ± SE, n=5).

| Species | C | N | P | Lignin | Cellulose | Phenol | C/N | Lignin/N |
| --- | --- | --- | --- | --- | --- | --- | --- | --- |
|  | (g kg^-1^) | (g kg^-1^) | (g kg^-1^) | (g kg^-1^) | (g kg^-1^ ) | (g kg^-1^) |  |  |
| *A. faxoniana* | 568.2 ± 26.2a | 17.26 ± 0.65a | 1.09 ± 0.06a | 149.5 ± 4.3a | 219.5 ± 9.2a | 22.67 ± 0.78a | 32.92 ± 2.15a | 12.72 ± 7.68a |
| *R. lapponicum* | 489.7 ± 4.0b | 8.34 ± 0.04b | 1.03 ± 0.03b | 181.8 ± 20.6b | 192.8 ± 49.5b | 23.50 ± 0.80a | 58.71 ± 3.10b | 23.12 ± 24.31b |

Different lowercase letters indicate a significant difference in a variable between species. Significance was evaluated at the *P* < 0.05 level.

Table S3 Abundance and percentages of principal soil faunal groups from litterbags of *A. faxoniana* litter decomposition in the three study sites.

|  | | | |  | |  |  | CF | |  | |  | |  | AS | |  | |  |  | AM |  |  |  | | Total | |  |
| --- | --- | --- | --- | --- | --- | --- | --- | --- | --- | --- | --- | --- | --- | --- | --- | --- | --- | --- | --- | --- | --- | --- | --- | --- | --- | --- | --- | --- |
|  | | | | Soil faunal group | |  | Number of individuals | Percentage  (%) | Abundance | |  | | Number of individuals | | Percentage  (%) | | Abundance | |  | Number of individuals | Percentage  (%) | Abundance |  | Number of individuals | Percentage  (%) | | Abundance | |
| Collembola | | | Poduridae | | |  | 82 | 9.92 | ++ | |  | | 100 | | 11.64 | | +++ | |  | 86 | 29.05 | +++ |  | 268 | 13.52 | | +++ | |
|  | | | Onychiuridae | | |  | 108 | 13.06 | +++ | |  | | 121 | | 14.09 | | +++ | |  | 11 | 3.72 | ++ |  | 240 | 12.11 | | +++ | |
|  | | | Sminthuridae | | |  | 2 | 0.24 | + | |  | |  | |  | |  | |  | 1 | 0.34 | + |  | 3 | 0.15 | | + | |
|  | | | Isotomidae | | |  | 194 | 23.46 | +++ | |  | | 79 | | 9.2 | | ++ | |  |  |  |  |  | 273 | 13.78 | | +++ | |
|  | | | Neanuridae | | |  | 26 | 3.14 | ++ | |  | | 8 | | 0.93 | | + | |  |  |  |  |  | 34 | 1.72 | | ++ | |
|  | | | Entomobryidae | |  |  | |  |  | |  | |  | |  | |  | |  | 1 | 0.34 | + |  | 1 | 0.05 | | + | |
| Prostigmata | | | Erythraeidae | | |  | 1 | 0.12 | + | |  | | 7 | | 0.81 | | + | |  | 12 | 4.05 | ++ |  | 20 | 1.01 | | ++ | |
|  | | | Pygmephoridae | | |  | 53 | 6.41 | ++ | |  | | 79 | | 9.2 | | ++ | |  | 14 | 4.73 | ++ |  | 146 | 7.37 | | ++ | |
|  |  |  | Rhagidiidae | | |  |  |  |  | |  | |  | |  | |  | |  | 1 | 0.34 | 0.05 |  | 1 | 0.05 | | + | |
|  | | | Stigmaeidae | | |  | 19 | 2.3 | ++ | |  | | 1 | | 0.12 | | + | |  | 4 | 1.35 | ++ |  | 24 | 1.21 | | ++ | |
|  | | | Cheylrtidae | | |  | 4 | 0.48 | + | |  | |  | |  | |  | |  |  |  |  |  | 4 | 0.2 | | + | |
| Mesostigmata | | | Parasitidae | | |  | 7 | 0.85 | + | |  | |  | |  | |  | |  | 2 | 0.68 | + |  | 9 | 0.45 | | + | |
|  | | | Laelapidae | | |  | 50 | 6.05 | ++ | |  | | 76 | | 9.15 | | ++ | |  | 71 | 23.99 | +++ |  | 197 | 9.94 | | ++ | |
|  | | | Ameroseiidae | | |  | 41 | 4.96 | ++ | |  | |  | |  | |  | |  | 20 | 6.76 | ++ |  | 61 | 3.08 | | ++ | |
|  | | | Ascidae | | |  | 2 | 0.24 | + | |  | |  | |  | |  | |  |  |  |  |  | 2 | 0.1 | | + | |
|  | | | Eriphididae | | |  |  |  |  | |  | |  | |  | |  | |  | 1 | 0.34 | + |  | 1 | 0.05 | | + | |
|  | | | Podocinidae | | |  | 9 | 1.09 | ++ | |  | | 4 | | 0.47 | | + | |  |  |  |  |  | 13 | 0.66 | | + | |
|  | | | Veigaiidae | | |  | 6 | 0.73 | + | |  | |  | |  | |  | |  |  |  |  |  | 6 | 0.3 | | + | |
|  | | | Parholaspididae | | |  | 69 | 8.34 | ++ | |  | | 98 | | 11.41 | | +++ | |  |  |  |  |  | 167 | 8.43 | | ++ | |
| Oribatid | | | Liacaridae | | |  | 71 | 8.59 | ++ | |  | | 85 | | 9.9 | | ++ | |  | 48 | 16.22 | +++ |  | 204 | 10.29 | | +++ | |
|  | | | Oribatuloidae | | |  | 10 | 1.21 | ++ | |  | | 42 | | 4.89 | | ++ | |  | 1 | 0.34 | + |  | 53 | 2.67 | | ++ | |
|  | | | Cepheus | | |  | 2 | 0.24 | + | |  | | 8 | | 0.93 | | + | |  |  |  |  |  | 10 | 0.5 | | + | |
|  | | | Lohmannidae | | |  |  |  |  | |  | | 2 | | 0.23 | | + | |  |  |  |  |  | 2 | 0.1 | | + | |
|  | | | Nothridae | | |  | 7 | 0.85 | + | |  | | 1 | | 0.12 | | + | |  | 3 | 1.01 | ++ |  | 11 | 0.55 | | + | |
|  | | | Palaeacaridae | | |  |  |  |  | |  | | 4 | | 0.47 | | + | |  |  |  |  |  | 4 | 0.2 | | + | |
|  | | | Galumnidae | | |  | 2 | 0.24 | + | |  | | 42 | | 4.89 | | ++ | |  | 6 | 2.02 | ++ |  | 50 | 2.52 | | ++ | |
| Diplura | | | Japygidae | | |  | 9 | 1.09 | ++ | |  | | 1 | | 0.12 | | + | |  |  |  |  |  | 10 | 0.5 | | + | |
|  | | | Campodae | | |  | 1 | 0.12 | + | |  | |  | |  | |  | |  |  |  |  |  | 1 | 0.05 | | + | |
| Diptera | | | Chironomidae | | |  | 27 | 3.26 | ++ | |  | | 72 | | 8.38 | | ++ | |  |  |  |  |  | 99 | 5 | | ++ | |
|  | | | Tipulidae | | |  | 6 | 0.73 | + | |  | | 2 | | 0.23 | | + | |  | 1 | 0.34 | + |  | 9 | 0.45 | | + | |
|  | | | Trichoceridae | | |  | 5 | 0.61 | + | |  | | 1 | | 0.12 | | + | |  |  |  |  |  | 6 | 0.3 | | + | |
|  | | | Dolichopodidae | | |  | 2 | 0.24 | + | |  | | 1 | | 0.12 | | + | |  |  |  |  |  | 3 | 0.15 | | + | |
|  | | | Anisopodidae | | |  | 2 | 0.24 | + | |  | | 8 | | 0.93 | | + | |  |  |  |  |  | 10 | 0.5 | | + | |
|  | | | Syrphidae | | |  |  |  |  | |  | | 1 | | 0.12 | | + | |  |  |  |  |  | 1 | 0.05 | | + | |
|  | | | Ptiliidae | | |  | 1 | 0.12 | + | |  | | 2 | | 0.23 | | + | |  |  |  |  |  | 3 | 0.15 | | + | |
|  | | | Elateridae | | |  |  |  |  | |  | |  | |  | |  | |  | 3 | 1.01 | ++ |  | 3 | 0.15 | | + | |
| Coleoptera | | | Staphylinidae | | |  | 3 | 0.36 | + | |  | | 2 | | 0.23 | | + | |  | 1 | 0.34 | + |  | 6 | 0.3 | | + | |
|  | | | Anthribidae | | |  |  |  |  | |  | |  | |  | |  | |  | 1 | 0.34 | + |  | 1 | 0.05 | | + | |
| Lepidoptera | | | Noctuidae | | |  |  |  |  | |  | |  | |  | |  | |  | 1 | 0.34 | + |  | 1 | 0.05 | | + | |
|  | | | Geometridae | | |  | 2 | 0.24 | + | |  | | 7 | | 0.81 | | + | |  | 1 | 0.34 | + |  | 10 | 0.5 | | + | |
| Araneae | | | Lycosidae | | |  | 1 | 0.12 | + | |  | |  | |  | |  | |  |  |  |  |  | 1 | 0.05 | | + | |
| Scorpiones | | | Selenopidae | | |  |  |  |  | |  | |  | |  | |  | |  | 1 | 0.34 | + |  | 1 | 0.05 | | + | |
| Psocoptera | | | Liposscelididae | | |  | 2 | 0.24 | + | |  | |  | |  | |  | |  |  |  |  |  | 2 | 0.1 | | + | |
|  | | | Elipsocidae | | |  | 1 | 0.12 | + | |  | |  | |  | |  | |  | 1 | 0.34 | + |  | 2 | 0.1 | | + | |
|  |  |  | Sphaeropsocidae | |  |  | |  |  | |  | | 4 | | 0.47 | | + | |  | 4 | 1.35 | ++ |  | 8 | 0.4 | | + | |
| Pseudoscorpionida | | | Cheliferidae | | |  |  |  |  | |  | | 1 | | 0.12 | | + | |  |  |  |  |  | 1 | 0.05 | | + | |
| Total | | |  | | |  | 827 |  |  | |  | | 859 | |  | |  | |  | 296 |  |  |  | 1982 |  | |  | |
| Number of groups | | |  | | |  | 34 |  |  | |  | | 29 | | |  | |  |  | 25 |  |  |  | 46 |  | |  | |

Note: +++: Dominant group (average density accounting for more than 10% of the total); ++: Frequent group (average density accounting for 1-10% of the total); +: Rare group (average density accounting for less than1% of the total).

Table S4 Abundance and percentages of principal soil faunal groups from litterbags of *R. lapponicum* litter decomposition in the three study sites.

|  | |  |  |  | CF | |  |  |  | | AS | |  |  |  | AM |  |  |  | | Total | |  |
| --- | --- | --- | --- | --- | --- | --- | --- | --- | --- | --- | --- | --- | --- | --- | --- | --- | --- | --- | --- | --- | --- | --- | --- |
|  | | Soil faunal group |  | Number of individuals | Percent  (%) | Abundance | |  | Number of individuals | | Percentage  (%) | | Abundance |  | Number of individuals | Percentage  (%) | Abundance |  | Number of individuals | Percentage  (%) | | Abundance | |
| Collembola | Poduridae | |  | 101 | 22.05 | +++ | |  | 69 | | 11.06 | | +++ |  | 46 | 25.27 | +++ |  | 216 | 17.09 | | +++ | |
|  | Onychiuridae | |  | 59 | 12.88 | +++ | |  | 95 | | 15.22 | | +++ |  | 7 | 3.85 | ++ |  | 161 | 12.74 | | +++ | |
|  | Sminthuidae | |  | 4 | 0.87 | + | |  |  | |  | |  |  |  |  |  |  | 4 | 0.32 | | + | |
|  | Isotomidae | |  | 36 | 7.86 | +++ | |  | 18 | | 2.88 | | ++ |  |  |  |  |  | 54 | 4.27 | | ++ | |
|  | Neanuridae | |  | 1 | 0.22 | + | |  |  | |  | |  |  | 6 | 3.3 | ++ |  | 7 | 0.55 | |  | |
| Prostigmata | Erythraeidae | |  |  |  |  | |  |  | |  | |  |  | 3 | 0.16 | + |  | 3 | 0.24 | | + | |
|  | Pygmephoridae | |  | 7 | 1.53 | ++ | |  | 19 | | 3.04 | | ++ |  | 16 | 8.79 | ++ |  | 42 | 3.32 | | ++ | |
|  | Bdellidae | |  | 1 | 0.22 | + | |  | 5 | | 0.8 | | + |  | 1 | 0.55 | + |  | 7 | 0.56 | | + | |
|  | Stigmaeidae | |  | 5 | 1.09 | ++ | |  | 10 | | 1.6 | | ++ |  |  |  |  |  | 15 | 1.19 | | ++ | |
| Mesostigmata | Parasitidae | |  | 2 | 0.44 | + | |  |  | |  | |  |  |  |  |  |  | 2 | 0.16 | | + | |
|  | Laelapidae | |  | 25 | 5.46 | ++ | |  | 28 | | 4.49 | | ++ |  | 28 | 15.38 | +++ |  | 81 | 6.41 | | ++ | |
|  | Ameroseiidae | |  | 30 | 6.55 | ++ | |  | 81 | | 12.98 | | +++ |  | 44 | 24.18 | +++ |  | 155 | 12.26 | | +++ | |
|  | Parholaspididae | |  | 23 | 5.02 | ++ | |  | 38 | | 6.09 | | ++ |  |  |  |  |  | 61 | 4.83 | | ++ | |
| Oribatid | Liacaridae | |  | 76 | 16.59 | +++ | |  | 162 | | 25.96 | | +++ |  | 3 | 1.65 | ++ |  | 241 | 19.07 | | +++ | |
|  | Oribatuloidae | |  |  |  |  | |  | 28 | | 4.49 | | ++ |  |  |  |  |  | 28 | 2.22 | | ++ | |
|  | Nothridae | |  | 29 | 6.33 | ++ | |  | 4 | | 0.64 | | + |  |  |  |  |  | 33 | 2.61 | | + | |
|  | Palaeacaridae | |  | 2 | 0.44 | + | |  | 3 | | 0.48 | | + |  |  |  |  |  | 5 | 0.4 | | + | |
|  | Galumnidae | |  | 3 | 0.66 | + | |  | 36 | | 5.77 | | ++ |  |  |  |  |  | 39 | 3.09 | | ++ | |
|  | Damaeidae | |  | 1 | 0.22 | + | |  | 2 | | 0.32 | |  |  |  |  |  |  | 3 | 0.24 | | + | |
|  | Lohmannidae | |  | 3 | 0.66 | + | |  | 2 | | 0.32 | | + |  |  |  |  |  | 5 | 0.4 | | + | |
| Diplura | Japygidae | |  | 5 | 1.09 | ++ | |  | 4 | | 0.64 | | + |  | 2 | 1.1 | ++ |  | 11 | 0.87 | | + | |
| Diptera | Chironomidae | |  | 19 | 4.15 | ++ | |  | 8 | | 1.28 | | ++ |  |  |  |  |  | 27 | 2.14 | | ++ | |
|  | Anisopodidae | |  | 9 | 1.97 | ++ | |  | 4 | | 0.64 | | + |  | 2 | 1.1 | ++ |  | 13 | 1.03 | | ++ | |
|  | Dolichopodidae | |  | 1 | 0.22 | + | |  | 1 | | 0.16 | | + |  | 2 | 1.1 | ++ |  | 4 | 0.32 | | + | |
| Coleoptera | Staphylinidae | |  | 3 | 0.66 | + | |  |  | |  | |  |  |  |  |  |  | 5 | 0.4 | | + | |
|  | Ptiliidae | |  | 2 | 0.44 | + | |  |  | |  | |  |  | 2 | 1.1 | ++ |  | 2 | 0.16 | | + | |
|  | Anthribidae | |  |  |  |  | |  |  | |  | |  |  | 2 | 1.1 | ++ |  | 2 | 0.16 | | + | |
| Lepidoptera | Noctuidae | |  |  |  |  | |  |  | |  | |  |  | 3 | 1.65 |  |  | 3 | 0.24 | | + | |
|  | Geometridae | |  | 1 | 0.22 | + | |  | 2 | | 0.48 | | + |  | 4 | 2.2 | ++ |  | 3 | 0.24 | | + | |
| Psocoptera | Liposscelididae | |  | 1 | 0.22 | + | |  |  | |  | |  |  | 4 | 2.2 | ++ |  | 5 | 0.3 | | + | |
|  | Sphaeropsocidae | |  | 2 | 0.44 | + | |  | 2 | | 0.32 | | + |  | 3 | 1.65 | ++ |  | 8 | 0.64 | | + | |
|  | Myopsocidae | |  | 1 | 0.22 | + | |  | 3 | | 0.48 | | + |  |  |  |  |  | 7 | 0.56 | | + | |
| Araneae | Lycosidae | |  | 1 | 0.22 | + | |  |  | |  | |  |  | 6 | 3.3 | ++ |  | 1 | 0.08 | | + | |
| Scorpiones | Selenopidae | |  |  |  |  | |  |  | |  | |  |  | 6 | 3.3 |  |  | 6 | 0.48 | | + | |
| Geolophilomorpha | Scolopendrellidae | |  | 4 | 0.88 | + | |  |  | |  | |  |  |  |  |  |  | 4 | 0.32 | | + | |
|  | Chilopoda | |  | 1 | 0.22 | + | |  |  | |  | |  |  |  |  |  |  | 1 | 0.08 | | + | |
| Total |  | |  | 458 |  |  | |  | 624 | |  | |  |  | 190 |  |  |  | 1272 |  | |  | |
| Number of groups |  | |  | 31 |  |  | |  | 23 |  | |  | |  | 17 |  |  |  | 37 |  | |  | |

Note: +++: Dominant group (average density accounting for more than 10% of the total); ++: Frequent group (average density accounting for 1-10% of the total); +: Rare group (average density accounting for less than1% of the total).
